# Comparison of Cr(VI) Adsorption Using Synthetic Schwertmannite Obtained by Fe^3+^ Hydrolysis and Fe^2+^ Oxidation: Kinetics, Isotherms and Adsorption Mechanism

**DOI:** 10.3390/ijms22158175

**Published:** 2021-07-30

**Authors:** Justyna Ulatowska, Łukasz Stala, Izabela Polowczyk

**Affiliations:** Department of Process Engineering and Technology of Polymer and Carbon Materials, Wroclaw University of Science and Technology, Wybrzeże Wyspiańskiego Street 27, 50-370 Wrocław, Poland; lukasz.stala@pwr.edu.pl (Ł.S.); izabela.polowczyk@pwr.edu.pl (I.P.)

**Keywords:** schwertmannite, Cr(VI), adsorption kinetics, isotherm models, pollution, iron-based sorbents

## Abstract

Good sorption properties and simple synthesis route make schwertmannite an increasingly popular adsorbent. In this work, the adsorption properties of synthetic schwertmannite towards Cr(VI) were investigated. This study aimed to compare the properties and sorption performance of adsorbents obtained by two methods: Fe^3+^ hydrolysis (SCH_A_) and Fe^2+^ oxidation (SCH_B_). To characterise the sorbents before and after Cr(VI) adsorption, specific surface area, particle size distribution, density, and zeta potential were determined. Additionally, optical micrographs, SEM, and FTIR analyses were performed. Adsorption experiments were performed in varying process conditions: pH, adsorbent dosage, contact time, and initial concentration. Adsorption isotherms were fitted by Freundlich, Langmuir, and Temkin models. Pseudo-first-order, pseudo-second-order, intraparticle diffusion, and liquid film diffusion models were used to fit the kinetics data. Linear regression was used to estimate the parameters of isotherm and kinetic models. The maximum adsorption capacity resulting from the fitted Langmuir isotherm is 42.97 and 17.54 mg·g^−1^ for SCH_A_ and SCH_B_. Results show that the adsorption kinetics follows the pseudo-second-order kinetic model. Both iron-based adsorbents are suitable for removing Cr(VI) ions from aqueous solutions. Characterisation of the adsorbents after adsorption suggests that Cr(VI) adsorption can be mainly attributed to ion exchange with SO_4_^2^^−^ groups.

## 1. Introduction

Environmental pollution is a constant struggle in our thriving society. Toxic metal ions present in certain process effluents, if discharged into surface water, are a threat not only to the environment [1,2] but directly to human health, as well [3]. The negative impact of toxic metal pollution in water on living organisms was described in the literature by many authors over the years [1,2]. Through decades, industry impact on the environment was ignored by governments, as in Poland or other Eastern Europe countries under Soviet Union influence [4]. This ignorance has led to irreversible devastation of the environment. Nowadays, emission problems are addressed in most regions of the world. Adequate institutions were established and set the emission limit values by the Best Available Techniques (European Commission, Directive 2010/75/EU). Those limit values can be lowered further as new techniques are developed, or the present methods are optimised to be more accessible and economically viable. There is a constant pursuit for better, cheaper, and safer toxic metal removal methods.

Chromium is one of many metals with ions that are considered toxic. Chromium mostly occurs in the two most stable oxidation states, Cr(III) and Cr(VI) [5], both of which are toxic to living organisms [6]. One of the most abundant elements on earth, chromium ions are present in the environment naturally deposited in the form of minerals [5]. However, chromium has found its use in many industry sectors, including metal processing or dye and pigment production [3]. Processing this metal leads to toxic chromium ions emissions into the environment with industrial wastewater. Another source of chromium contamination is fumes from fossil fuel incineration [3]. Chromium released into the environment in excess through anthropogenic activity is threatening to the environment and human health. Hence, there is a need to improve existing chromium ion removal techniques to minimise human-caused devastation. 

Over the past few decades, water treatment became one of the most pressing industry matters. Many metal ion removal techniques were developed. Chromium ion removal techniques include adsorption [7], biosorption [8], chemical precipitation [9], ion exchange [10], and electrochemical methods [11]. Adsorptive methods can be very efficient and employ low-cost materials-by-products or waste products of certain large-scale processes. Fly ash would be an example, as certain fly ash types can perform well as adsorbents on their own or after brief modification [12,13,14]. Use of problematic waste such as fly ash or iron oxides to remove toxic ions fulfils the guidelines of sustainable management and development.

Iron oxyhydroxides are known adsorbents of toxic chromium ions as weak acid oxyanions have a strong affinity towards their proton-specific surface sites [15]. Iron-containing minerals can effectively remove chromium oxyanions in acidic conditions (pH 2–7). However, oxyanions affinity towards iron oxyhydroxides can be disrupted by other ion species such as sulphuric, selenite, or phosphate anions [16,17]. Adsorption is one of two mechanisms employed by iron oxyhydroxides as chromium ion removal agents. The second mechanism of Cr(VI) removal from aqueous solutions is their reduction with the use of iron(II)-containing minerals [18,19], as the products of the reduction are poorly soluble in water. New materials based on chromium affinity towards iron-containing minerals are still being developed. Researchers have published reports on novel solutions implementing iron oxides and oxyhydroxides properties [20,21].

Schwertmannite is a name of an iron–oxyhydroxysulfate mineral with a chemical formula of Fe^3+^_16_O_16_(OH,SO_4_)_12__−__13_ × 10^−^^12^ H_2_O. It is chemically stable, although it tends to transform into goethite in acidic conditions [22]. Schwertmannite loaded with Cr(VI) oxyanions is stable at pH 4 and does not transform to goethite [22]. Schwertmannite occurs naturally, but for pollutant removal, synthetic schwertmannite is usually used [23]. Schwertmannite sorbents can remove a variety of contaminants including fluorides, toxic metal ions, oxyanions, and organic pollutants [23,24,25,26,27,28,29,30,31,32]. Schwertmannite is an excellent oxyanion adsorbent as it is characterised by positive surface potential and a large surface area of 100–200 m^2^·g^−1^, and additionally its crystallographic structure allows it to incorporate oxyanions to its crystal structure [22]. Schwertmannite is a universal adsorbent that requires careful investigation of its adsorptive properties. Synthetic schwertmannite is attracting interest because, due to its simple and relatively inexpensive method of production, it could provide an alternative to natural schwertmannite, thereby reducing its exploitation from the environment.

This paper presents adsorption studies of Cr(VI) on synthetic schwertmannite sorbents obtained through two different synthesis methods: Fe^2+^ oxidation and Fe^3+^ hydrolysis. The physicochemical characteristics of the sorbents were determined, described, and then contrasted. The adsorption properties of two sorbents were then compared in terms of Cr(VI) affinity under varying conditions of dosage, pH, time, and Cr(VI) concentration. The effects of pH and dosage were investigated to find the optimum process parameters and then kinetics and isotherm experiments were performed and described.

## 2. Results and Discussion

### 2.1. Characterisation of Adsorbents

**Figure 1:**
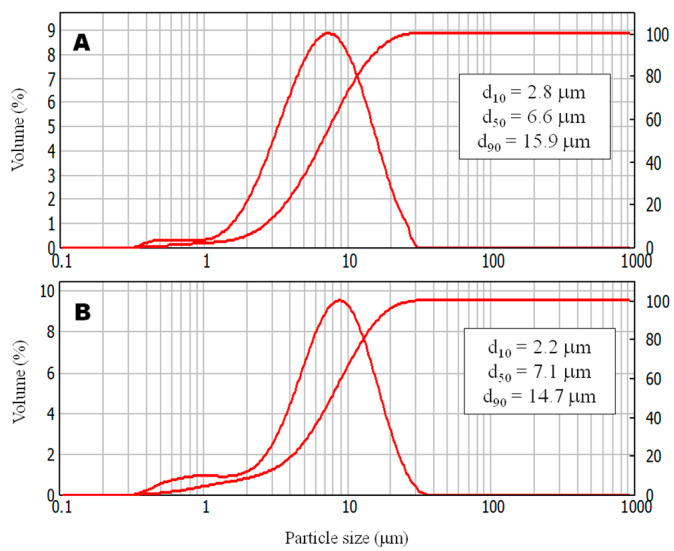
The particle size distribution of obtained schwertmannite: (**A**) SCH_A_, (**B**) SCH_B_.

The particle size analysis by laser diffraction enabled visualisation of the volumetric particle size distribution of the synthesised schwertmannite by two methods: Fe^3+^ hydrolysis (SCH_A_) and Fe^2+^ oxidation (SCH_B_). The particle size distributions for both materials are shown in Figure 1.

A complete analysis of particle size by laser diffraction showed that the particle size distribution of SCH_A_ indicated a median diameter (d_50_) of about 6.6 μm, while d_10_ and d_90_ were 2.8 and 15.9 μm, respectively. SCH_B_ particle size analysis showed a median diameter (d_50_) of approximately 7.1 μm, while d_10_ and d_90_ were 2.2 and 14.7 μm, respectively. It can be seen in Figure 1 that the systems considered are dominated by particles in the range of 2 to 15 μm, but a group of particles smaller than 1 μm also appears, suggesting that the resulting materials may be nano-sized. Additionally, a parameter, which shows the width of the size distribution (span), was calculated. The span is determined as [33]:

span = (d_90_ − d_10_)/d_50_ (1)

The span indicates how far the 10% and 90% points are apart, normalised with the midpoint. Merkus, in his classification, proposed six ranges of the width of the size distribution [33]: span < 1.02 dimensional, 1.02 < span < 1.05 very narrow, 1.05 < span < 1.5 narrow, 1.5 < span < 4.0 average, 4.0 < span < 10 wide, and span > 10 very wide. The calculated using Equation (1) span values for SCH_A_ and SCH_B_ are 1.98 and 1.76, respectively, which means that the width of the particle size distribution for tested schwertmannite sorbents is in the average range. 

**Figure 2:**
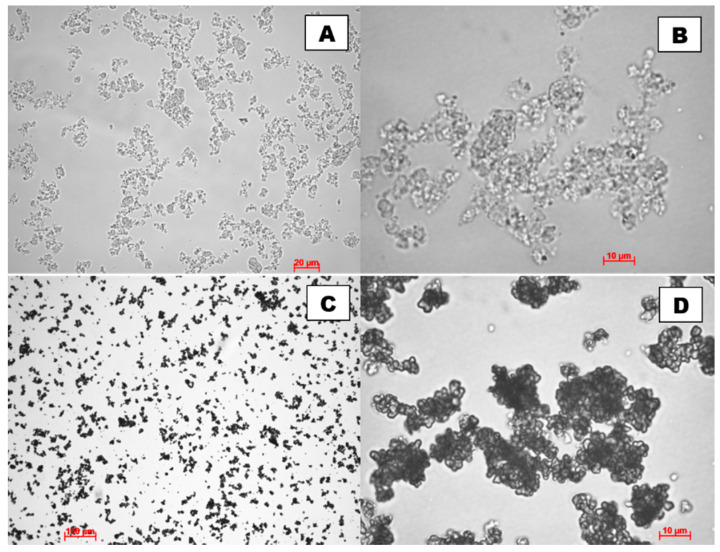
Images of synthetic schwertmannite samples taken using an optical microscope: (**A**,**B**) are SCH_A_; (**C**,**D**) are SCH_B_.

**Figure 3:**
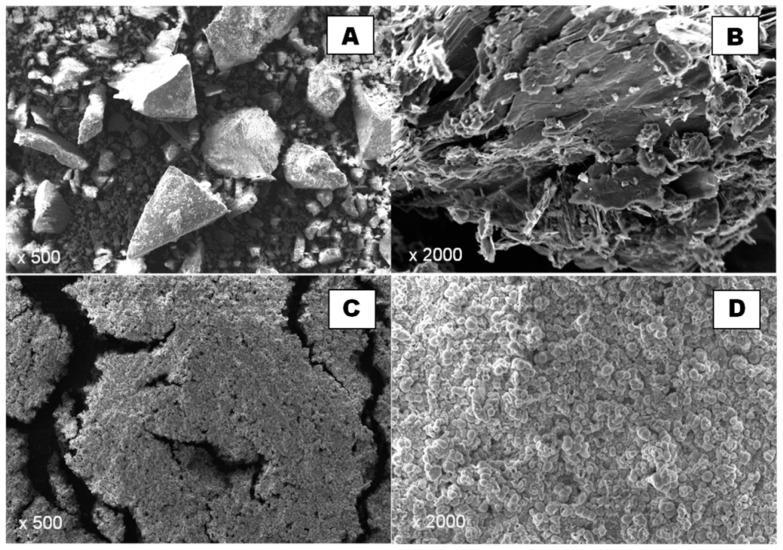
Images of synthetic schwertmannite samples taken with a scanning electron microscope: (**A**,**B**) are SEM images of SCH_A_; (**C**,**D**) are SEM images of SCH_B_.

The morphology and particle size of the produced schwertmannite were observed using both optical and scanning electron microscope. The optical micrographs are shown in Figure 2 and SEM micrographs in Figure 3. The size of adsorbent particles is visible in the optical microscope images. Particles of several hundred nanometres in size are visible alongside aggregates of around a few tens of microns, as confirmed by an earlier analysis of the particle size distribution (Figure 1). The SCH_A_ sample is brighter in transmitted light, and has more crystalline and smaller particles than the SCH_B_ sample.

In the SCH_A_ sample (Fe^3+^ hydrolysis), disorderly distributed, elongated, locally fibrous crystallites forming clusters with considerable porosity can be observed (Figure 3A,B). In contrast, characteristic “hedgehog” aggregates can be observed in the SCH_B_ sample (Fe^2+^ oxidation), but the main forms in which schwertmannite occurs are rounded, isomeric aggregates of about 2 μm in size (Figure 3C,D), resembling ferrihydrite clusters but differing in surface character, indicating that they consist of very fine, elongated crystallites. Very similar morphological features were observed for synthetic schwertmannite in the literature [27,34,35,36,37,38,39,40,41,42,43]. Finally, the SEM images show the size of the sorbent particles produced.

The extent of the specific surface area of the sorbents is strongly influenced by the surface morphology and schwertmannite particle size. The measured BET specific surface area was 78.6 and 136.7 m^2^·g^−1^ for SCH_A_ and SCH_B_, respectively. The well-developed specific surface area suggests that both obtained schwertmannite samples may be good Cr(VI) sorbents. The literature also confirms that schwertmannite obtained by chemical oxidation is characterised by a smaller surface area than that obtained by rapid hydrolysis. Chemical oxidation yields schwertmannite with a specific surface area of 4–14 m^2^·g^−^^1^ [30], 2.06–16.3 m^2^·g^−^^1^ [44], 48.2 m^2^·g^−^^1^ [27], and even 130.9 m^2^·g^−^^1^ [45]. In contrast, rapid hydrolysis enables the synthesis of schwertmannite with a specific surface area several times greater, e.g., 165 m^2^·g^−^^1^ [46], 199.4 m^2^·g^−^^1^ [47], 206.1 m^2^·g^−^^1^ [29], and even 325.5 m^2^·g^−^^1^ [48]. The density of SCH_A_ and SCH_B_ was determined using a pycnometer, and amounts to 3.54 and 3.75 g·cm^−^^3^, respectively.

**Figure 4:**
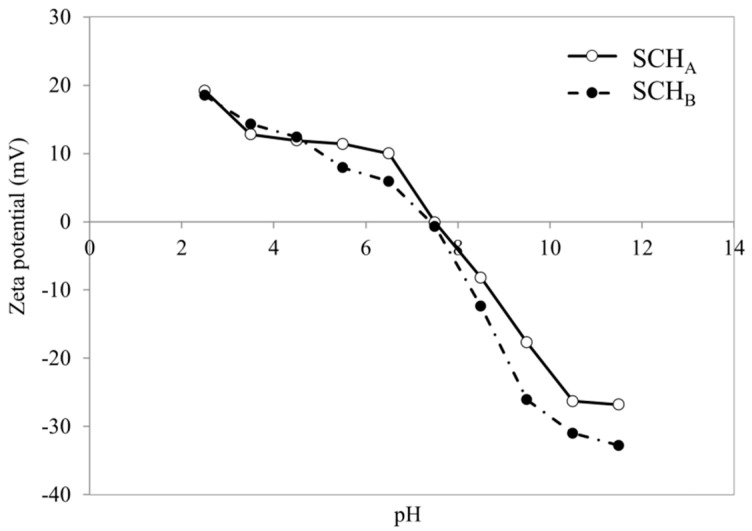
Zeta potential of synthetic schwertmannite adsorbents (SCH_A_ and SCH_B_) as function of pH.

Figure 4 shows the zeta potential changes in the pH function for schwertmannite sorbents (SCH_A_ and SCH_B_). Zeta potential measurements are essential to determine the surface potential of these adsorbents. The results showed that the negative charge and thus the negative potential increases with increasing pH and reaches a pH 11.5 maximum value of −26.8 and −32.8 mV for SCH_A_ and SCH_B_, respectively. At acidic pH, the zeta potential of the tested adsorbents is positive, and at pH 4.5 it takes the value of +11.9 and +12.4 mV for SCH_A_ and SCH_B_, respectively. As reported by Cornell and Schwertmann, iron minerals usually exhibit so-called points of zero charge at neutral pH and therefore are often used as anion and cation adsorbents [49]. For both synthetic schwertmannite adsorbents, the isoelectric point is observed at pH_iep_ around 7.5. The obtained data are consistent with the literature, as some authors report that the isoelectric point of schwertmannite is in the range 5.4–7.4 [40,49].

### 2.2. Effect of Adsorbent Dose

**Figure 5:**
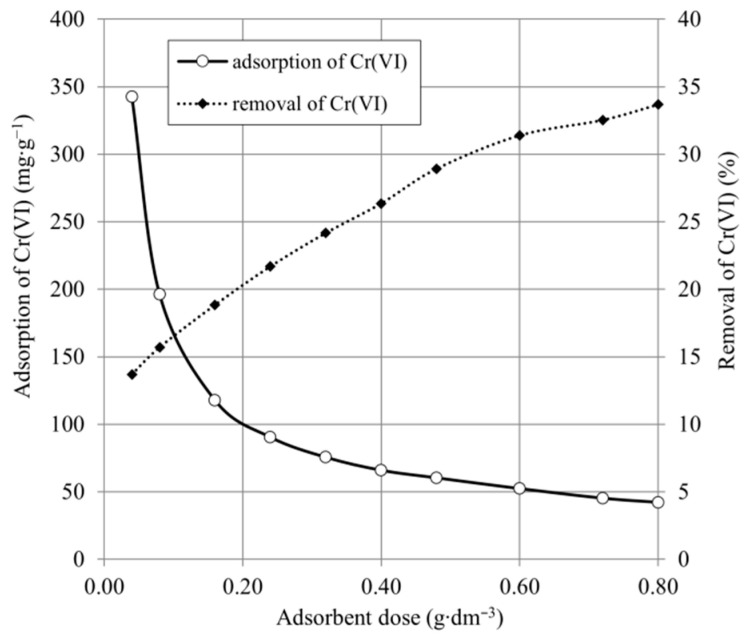
The adsorption capacity and Cr(VI) removal of SCH_A_ (100 mg·dm^−^^3^; 25 °C; pH 4.5).

**Figure 6:**
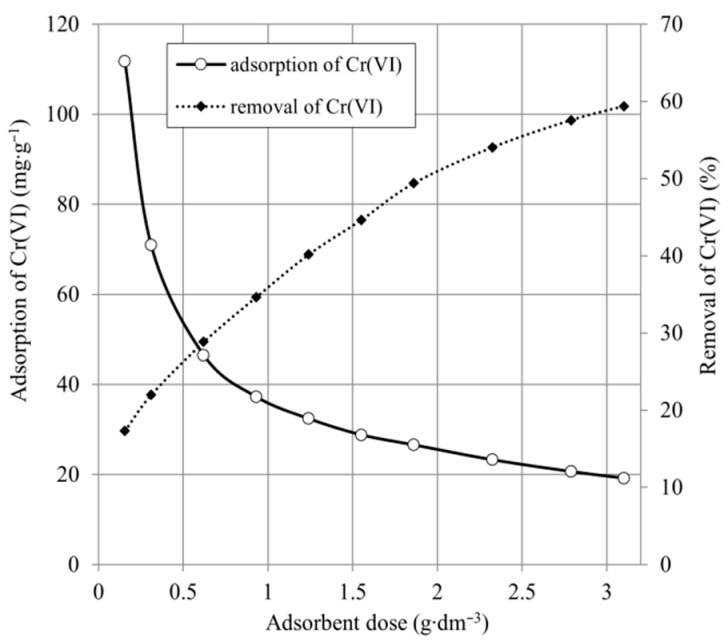
The adsorption capacity and Cr(VI) removal of SCH_B_ (100 mg·dm^−^^3^; 25 °C; pH 4.5).

The effect of the adsorbent dose is an important parameter determining the removal efficiency and the economics of the process. Figure 5 and Figure 6 show the influence of adsorbent dose on Cr(VI) removal.

As shown in Figure 5, the adsorption capacity decreased from 324.5 to 42.1 mg·g^−^^1^, and the percentage of Cr(VI) removal increased from 13.7% to 33.9%, proportionally to the increase in SCH_A_ dose. The experimental results in Figure 5 show that the adsorption capacity decreased from 111.7 to 19.2 mg·g^−^^1^ and the percentage of Cr(VI) removal increased from 17.3% to 59.4%, in proportion to the increase in SCH_B_ dose. The decrease in adsorption capacity with increasing adsorbent dose is due to the fact that the more active adsorbent sites remain unsaturated during the adsorption process. The maximum static uptake of Cr(VI) by the SCH_A_ and SCH_B_ samples was achieved for minimal possible adsorbent dose and was 342.5 and 111.7 mg·g^−^^1^, respectively. The maximum Cr(VI) reduction was obtained for maximal adsorbent dose and was 33.6% and 59.4%, respectively, for SCH_A_ and SCH_B_. The results clearly indicate that despite the lower dry matter content of the SCH_A_ adsorbent, the Cr(VI) uptake in this adsorbent is significantly higher than of SCH_B_.

### 2.3. Effect of pH

**Figure 7:**
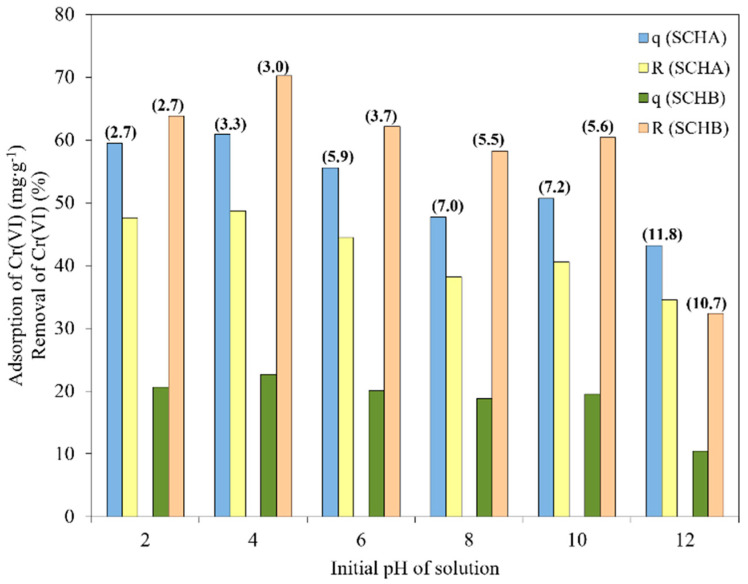
Effect of pH on adsorption and removal of Cr(VI) onto SCH_A_ and SCH_B_ (q is adsorption of Cr(VI) (mg·g^−^^1^), R is removal of Cr(VI) (%); the final pH values are shown above the bar graphs) (100 mg·dm^−^^3^; 25 °C; volume of adsorbent 1 cm^3^).

SCH_A_ and SCH_B_ samples were contacted with solutions of pH varying from 2 to 12. The effect of pH on Cr(VI) adsorption and removal is shown in Figure 7. 

As can be seen in Figure 7, Cr(VI) adsorption is the highest for acidic solutions, and Cr(VI) adsorption decreases with increasing pH. The optimum pH for Cr(VI) removal was set at 4.0 and a maximum Cr(VI) adsorption capacity of 60.9 and 22.7 mg·g^−^^1^ was obtained for SCH_A_ and SCH_B_, respectively. The lowest removal ratio was observed at pH 12. Additionally, it can be seen that the pH of the solution changed after adsorption. SCH_A_ and SCH_B_ have a pH of about 5.5, and the addition of these materials caused the pH of the solutions to change after adsorption. For solutions with an initial acidic pH, an increase in pH is observed, while a decrease in pH is observed for basic solutions. The effect of pH on the adsorption of Cr(VI) on schwertmannite can be attributed to the chromium speciation and surface charge of the adsorbent in an aqueous solution. Cr(VI) can occur in aqueous solutions as chromate (CrO_4_^2^^−^), dichromate (Cr_2_O_7_^2^^−^), hydrogen chromate (CrHO_4_^−^), and hydrogen dichromate (Cr_2_HO_7_^−^) ions, as well as chromic acid. In strongly acidic solutions (pH < 3) it exists only as HCrO_4_^−^ and CrO_7_^2^^−^ ions. Chromate ions are predominant at pH > 7, whereas hydrogen chromate ions are predominant at pH < 6 [50]. According to Anah and Astrini, high adsorption in acidic conditions can be explained by the fact that negatively charged Cr(VI) ions are strongly attracted to the positively charged adsorbent surface [51]. As can be seen from the zeta potential study (Figure 4), at alkaline pH, the surface potential of schwertmannite is negative and significantly, resulting in lower adsorption of Cr(VI) ions. When the zeta potential of adsorbent particles is negative, the electrostatic attraction between Cr(VI) ions and the schwertmannite surface decreases, leading to a reduced adsorption capacity. On the other hand, the zeta potential value becomes positive at acidic pH, increasing the electrostatic force between Cr(VI) ions and the schwertmannite surface. Thus, the adsorption capacity increases. Additionally, at alkaline pH conditions, inhibition of Cr(VI) hydrolysis may be one of the reasons for reduced adsorption [51]. Zhang and co-workers reported that an increase in pH may also release sulphate groups through higher concentration of OH^−^ ions, converting schwertmannite to goethite, and this may decrease the adsorption efficiency of Cr(VI) [52].

Since the most significant amount of Cr(VI) ions was adsorbed from a solution with an initial pH of 4.5, all adsorption experiments were conducted at pH 4.5.

### 2.4. Effect of Contact Time

**Figure 8:**
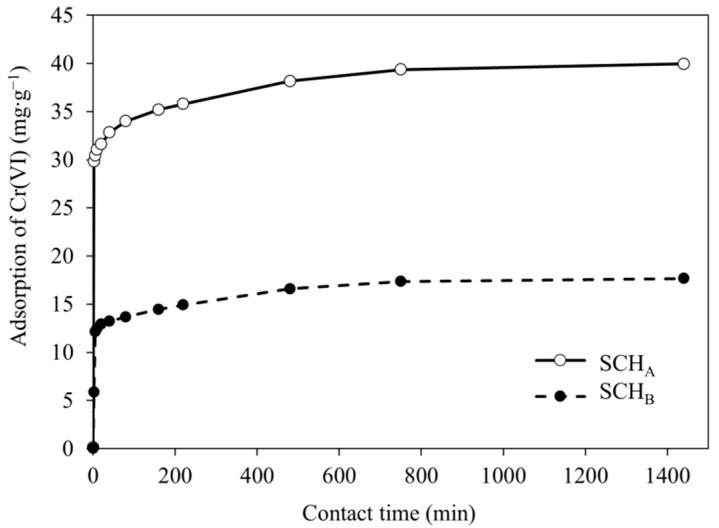
Effect of contact time on adsorption of Cr(VI) (100 mg·dm^−^^3^; 25 °C; pH 4.5; volume of adsorbent 1 cm^3^).

The effect of contact time on the amount of Cr(VI) ions removal from the solution during the adsorption by schwertmannite sorbents (SCH_A_ and SCH_B_) was studied with the use of the solution containing 100 mg·dm^−^^3^ of Cr(VI). The solution with added adsorbent was stirred over 24 h and the samples were drawn periodically to measure the residual Cr(VI) concentration starting from the second minute at room temperature (25 ± 1 °C). Figure 8 shows the effect of contact time on the adsorption of Cr(VI).

The equilibrium for both SCH_A_ and SCH_B_ adsorbents was achieved after 12 h. No significant changes in Cr(VI) concentration in solution were observed after equilibrium was reached. The results showed that Cr(VI) adsorption process on schwertmannite increases with increasing contact time until an equilibrium is attained between Cr(VI) ions adsorbed on the schwertmannite surface and Cr(VI) ions present solution. It can also be observed that Cr(VI) adsorption on both schwertmannite sorbents was faster in the first minutes of the process and gradually slowed down until equilibrium was reached. This effect is related to the saturation of the available surface-active sites in the initial stages of the adsorption process. Figure 8 also shows that more Cr(VI) ions were adsorbed on SCH_A_ than on SCH_B_.

#### Adsorption Kinetic Models

To investigate the mechanism and determine the rate-controlling step in the adsorption of Cr(VI) ions onto synthetic schwertmannite sorbents, kinetic models were used. The rate constants were calculated using pseudo-first-order (PFO) (Equation (2)) and pseudo-second-order (PSO) kinetic models (Equation (3)) [53,54,55,56], while the rate-controlling step was determined using the intraparticle diffusion (IPD) (Equation (4)) and the liquid film diffusion (LFD) models (Equation (5)) [57,58,59]. Table 1 shows the equations of the applied models.

It is observed that the adsorption kinetics data fit both models well (R^2^ > 0.9), but judging by high R^2^ values, the adsorption data were better fitted by the PSO model. The experimental and calculated adsorption capacity (q_e_) values of the PSO model indicate better consistency than the PFO model, as evidenced by significantly lower standard deviation (SD) values. The calculated adsorption capacities (q_2_) by the PSO model are close to their experimental values (q_exp_) both for SCH_A_ and for SCH_B_ (Table 2). These results prompt that the adsorption of Cr(VI) onto SCH_A_ and SCH_B_ schwertmannite sorbents follows the PSO kinetic, further suggesting that chemisorption is the rate-controlling step [55]. The PSO rate constants were calculated as 2.7 × 10^−3^ and 0.7 × 10^−3^ g·mg^−1^·min^−1^ for SCH_A_ and SCH_B_, respectively. The PSO rate constant is over three times greater for SCH_A_. This is directly correlated with the significant difference between initial adsorption rates, which are 4.18 and 0.228 mg·g^−1^·min^−1^ for SCH_A_ and SCH_B_, respectively. The obtained initial adsorption rates indicate that Cr(VI) adsorption on SCH_A_ is faster than on SCH_B_.

In addition, it was investigated whether intraparticle diffusion (IPD) or liquid film diffusion (LFD) kinetics play an important role in Cr(VI) adsorption from aqueous solutions on schwertmannite sorbents. Table 3 summarises the values of the calculated parameters for IPD and LFD models, and gives the values of the determination coefficient (R^2^).

**Figure 9:**
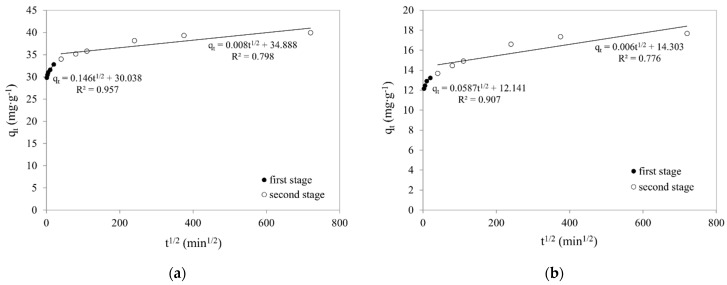
Intraparticle diffusion model of Cr(VI) adsorption on schwertmannite sorbents: (**a**) SCH_A_; (**b**) SCH_B_ (100 mg·dm^−3^; 25 °C; pH 4.5; volume of adsorbent 1 cm^3^).

The IPD rate was obtained from the plots of q_t_ vs t^1/2^. In theory, the slope of this plot can be linearised in four regions, which represent the distinct stages of mass transfer of adsorbates onto adsorbents. The initial stage represents external mass transfer with the following three stages being intraparticle diffusion in the macro-, meso-, and micropore structure of the adsorbent [60,61]. As can be seen in Figure 9 (the linear dependence of q_t_ to t^1/2^), two stages of diffusion can be distinguished, both for the adsorption of Cr(VI) on SCH_A_ and on SCH_B_. Additionally, the first segment does not pass through the origin of the coordinate system (parameter B is different from zero), suggesting that internal diffusion is not the only limiting step in the liquid phase adsorption process in this study. It is said that the greater the value of B, the greater the influence of the boundary layer on the adsorption process. As a result of the linear extrapolation of the first stage and the intersection of this line with the ordinate axis, a value corresponding to diffusion in the boundary layer was obtained, while the second linear part concerns diffusion inside the pores. The values of k_IPD1_, k_IPD2_, and B were calculated from the slope of each line. The obtained values are given in Table 3. If the value of k_IPD1_ is greater than the value of k_IPD2_, it indicates that the boundary layer controls the adsorption of Cr(VI) on schwertmannite sorbents.

According to the obtained results, suggesting that adsorption in the boundary layer is the limiting stage of the adsorption process, the effectiveness of fitting the experimental data to the liquid film model was also investigated. A linear plot of ln(1 − (q_t_/q)) vs. t, with zero intercepts, would suggest that the kinetics of the adsorption process is controlled by liquid film diffusion.

**Figure 10:**
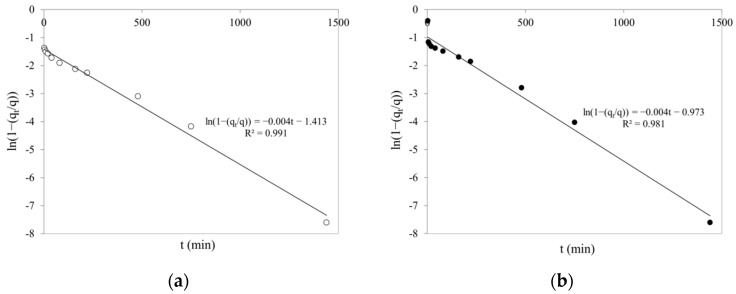
Liquid film diffusion model of Cr(VI) adsorption on schwertmannite sorbents: (**a**) SCH_A_; (**b**) SCH_B_ (100 mg·dm^−3^; 25 °C; pH 4.5; volume of adsorbent 1 cm^3^).

As can be seen in Figure 10, the plots are linear but do not pass through the coordinate origin. Thus, diffusion in the liquid layer is not the dominant mechanism of Cr(VI) adsorption on SCH_A_ and SCH_B_ schwertmannite sorbents. The rate constant for liquid film diffusion (k_LFD_) was 0.0041 and 0.0044 min^−1^ for SCH_A_ and SCH_B_, respectively (Table 3).

### 2.5. Effect of Initial Cr(VI) Concentration

**Figure 11:**
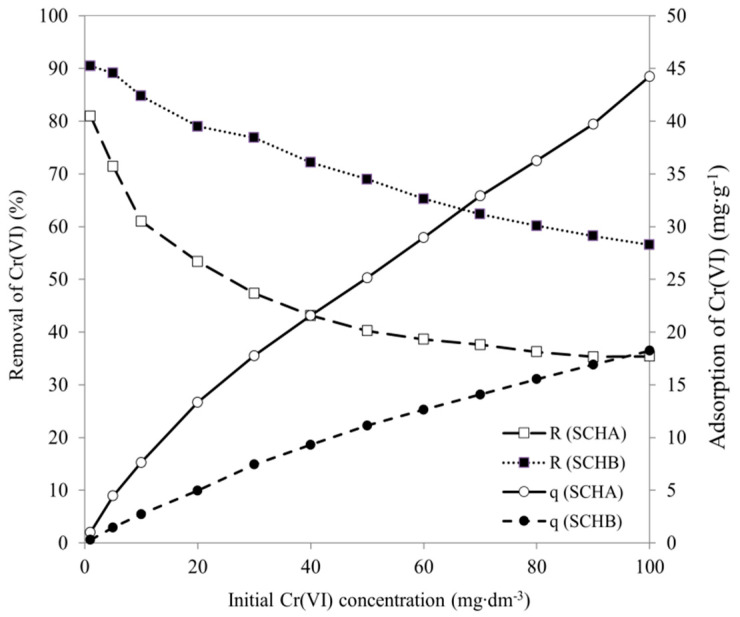
Effect of initial Cr(VI) concentration (R is removal of Cr(VI) (%), q is adsorption of Cr(VI) (mg·g^−^^1^)) (25 °C; pH 4.5; volume of adsorbent 1 cm^3^).

The effect of initial Cr(VI) concentration on adsorption onto SCH_A_ and SCH_B_ is presented in Figure 11. The results show that as the initial concentration increases, the amount of adsorbed Cr(VI) ions increases but the removal rate decreases. At higher initial concentration, the active sites of adsorbent are surrounded by more Cr(VI) ions in solution. Therefore, the equilibrium adsorption capacity increases with increasing initial Cr(VI) concentration. Furthermore, the percentage of Cr(VI) removal decreases with an increase in the initial Cr(VI) concentration. At low initial concentration, the ratio of the initial number of Cr(VI) ions to available active sites of adsorbent is low. Therefore, the percentage removal of Cr(VI) is higher, and at higher concentration, further residual Cr(VI) ions remain in solution [62].

#### Adsorption Isotherm Models

Estimation of the adsorption isotherm is crucial to determine the adsorption capacity and to study the adsorption nature. In this work, the experimental data on Cr(VI) adsorption equilibrium have been investigated using three two-parameter equations: Freundlich (Equation (6)) [63,64], Langmuir (Equation (7)) [64,65], and Temkin (Equation (8)) [64,66]. Table 4 shows the equations of the applied isotherm models.

The parameters of adsorption isotherms were estimated by linear regression method. In order to determine the parameters by linear regression, the adsorption isotherm equations are transformed to the linearised forms. The isotherm parameters were estimated based on the slope of the regression line and the intersection of the straight line with the axis of ordinates. The criterion for fitting the model was the value of the coefficient of determination (R^2^).

The adsorption isotherm parameters were determined for both measurement series, in the range of low and high initial Cr(VI) concentrations. The calculated parameters are presented in Table 5.

It can be seen in Table 5 that the Freundlich isotherm model best describes the experimental data—the values of the coefficient of determination close to 1. The Freundlich model is the best for describing data in the low concentration range (1–100 mg·dm^−^^1^), which is confirmed by numerous literature sources [63,64]. On the other hand, in the wide range of initial concentrations studied, the Freundlich model performs well for Cr(VI) adsorption on SCH_A_. At the same time, the Langmuir isotherm model also performs well for adsorption on SCH_B_. The Temkin isotherm model fails when describing the adsorption of Cr(VI) on synthetic schwertmannite (R^2^ < 0.85). Therefore, it is not discussed in detail in this paper.

The dimensionless parameter 1/n in the Freundlich equation allows determining the adsorption intensity or surface heterogeneity indicating. When 1/n is greater than zero (0 < 1/n < 1), the adsorption is favourable; when 1/n is greater than 1, the adsorption process is unfavourable; and it is irreversible when 1/n = 1 [67]. The calculated values based on static adsorption tests are in the range of 0 < 1/n < 1, suggesting that Cr(VI) adsorption on schwertmannite is a favourable process. From the values of 1/n, it can also be concluded that the adsorbent surface is partially heterogeneous, as the inverse of the parameter n is closer to 1 than to 0. Schwertmannite is a versatile adsorbent that requires careful study of its adsorption properties. The k_F_ parameter, on the other hand, is defined as the capacitance factor, which depends on 1/n. The obtained values for k_F_ are higher for SCH_A_ than for SCH_B_.

**Figure 12:**
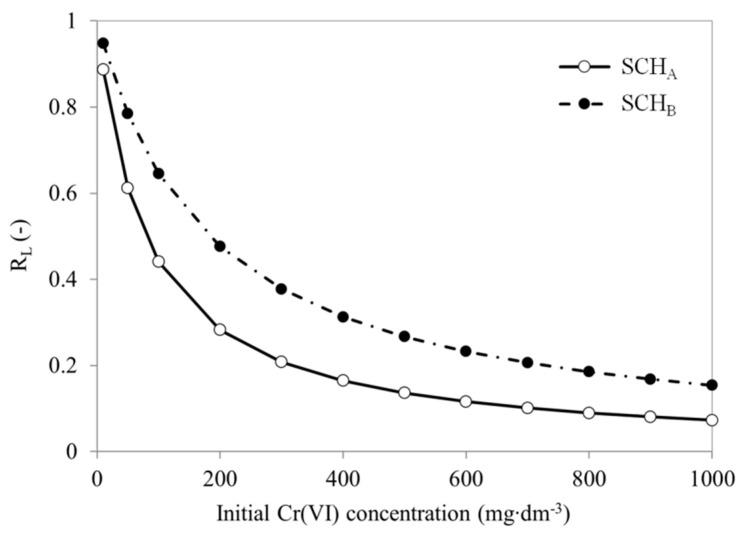
The calculated separation coefficient profile for Cr(VI) as a function of the initial Cr(VI) concentration.

The validity of the Langmuir isotherm model was checked by calculating the separation coefficient R_L_ [68,69]. It can be calculated from the following equation (Equation (9)) [70]:

R_L_ = 1/(1 + k_L_·c_0_) (9)

where k_L_ and q_L_ are parameters of Langmuir isotherm. As shown in Figure 12, the ranges of values of the separation coefficient R_L_ were in all cases between 0 and 1 range, which means that the adsorption under the studied conditions was favourable [64,70].

Figure 12 shows the dependence of the equilibrium parameter on the initial concentration of Cr(VI) solution. The R_L_ values range from 0 to 1, suggesting that adsorption proceeds favourably and SCH_A_ and SCH_B_ are suitable adsorbents for removing Cr(VI) ions from aqueous solutions.

Considering the high value of the coefficient of determination for the Langmuir isotherm (R^2^ > 0.96), it can be assumed that the calculated maximum adsorption capacity towards Cr (VI) at a low initial concentration of this element in the solution can reached 42.97 and 17.54 mg·g^−^^1^ for SCH_A_ and SCH_B_, respectively, which is in agreement with Figure 5 and Figure 6. For higher initial concentrations of Cr(VI), on the other hand, it can achieve 201.8 and 131.8 mg·g^−^^1^ for SCH_A_ and SCH_B_, respectively. According to Zhang and co-workers and Gan and co-workers, Freundlich and Langmuir models are the best fitting models for adsorption of Cr(VI) on schwertmannite [26,52], confirmed by the obtained experimental results.

### 2.6. Comparison with Other Studies

Cr(VI) removal on schwertmannite sorbents has been carried out and described by few authors. The literature survey shows that there are three different synthesis methods that yield schwertmannite of varying quality. These methods are Fe^2+^ oxidation, Fe^3+^ hydrolysis, and biosynthesis. However, there is still insufficient data to establish which synthesis method is superior for Cr(VI) removal, as most of the papers on schwertmannite adsorbents explore the affinity towards arsenic oxyanions for these hydroxysulphates [24,27,29,37,71]. The maximum adsorption capacities of schwertmannite sorbents synthesised by these three methods towards Cr(VI) are presented in Table 6. Bio-synthesised schwertmannite sorbents reach capacities up to 58.2 mg·g^−^^1^ towards Cr(VI). In contrast, the adsorption capacities of chemically synthesised schwertmannite sorbents vary significantly from 17.5 mg·g^−^^1^ to 219 mg·g^−^^1^ for materials obtained by Fe^2+^ oxidation and from 43.0 mg·g^−^^1^ to 178.6 mg·g^−^^1^ for materials obtained by Fe^3+^ hydrolysis. The synthesis of mineral sorbents can be influenced by small changes in reaction conditions, including temperature, pressure, and concentration of substrates. Therefore, method optimisation is an important part of sorbent design.

As the sorbents reported in the literature show promising adsorption properties, including large surface area and favourable surface potential, it is important to continue research and gain further data on the schwertmannite synthesis process to obtain the most efficient product. An important conclusion is that the adsorption properties of schwertmannite are affected by the method of fabrication of this material. Despite their formal structural similarity, SCH_A_ and SCH_B_ have different characteristics that affect their adsorption capacity towards Cr(VI). SCH_A_ has a smaller specific surface area but has a higher adsorption capacity. It is related to the structure of this adsorbent. It is finer and formed by fibrous crystallites (Figure 3B), which adsorb Cr(VI) better. In contrast, SCH_B_ is mainly rounded aggregates (Figure 3D) that give a larger specific surface area. Still, their arrangement probably makes it difficult to access all active sites, and a lower adsorption capacity of this material is observed.

### 2.7. Possible Cr(VI) Adsorption Mechanism

As reported by several authors, Cr(VI) adsorption on schwertmannite can be mainly attributed to ion exchange with SO_4_^2^^−^ groups [22,25,52,71]. Due to the same charge and a similar radius of SO_4_^2^^−^ (0.23 nm) and CrO_4_^2^^−^ (0.24 nm) ions, the replacement of sulphate groups with chromate groups occurs without changing the crystal structure of the schwertmannite [52]. No change in the structure of synthetic schwertmannite is observable when analysing FTIR spectra before and after adsorption of Cr(VI) and SEM images of schwertmannite after adsorption of Cr(VI).

According to the literature, the schwertmannite spectrum can consist of seven to eleven characteristic active infrared bands. Five of them correspond to sulphate vibrations and are located at wavenumber about 610, 975, 1055, 1130, and 1200 cm^−^^1^ [22,23]. In addition, bands in the 3300 cm^−^^1^ and 1630 cm^−^^1^ range originate from the vibrations of the −OH and H_2_O groups, respectively. In the range of 400–700 cm^−1^, stretching bands appear originating from FeO bonds [23,34,52,75,76]. 

**Figure 13:**
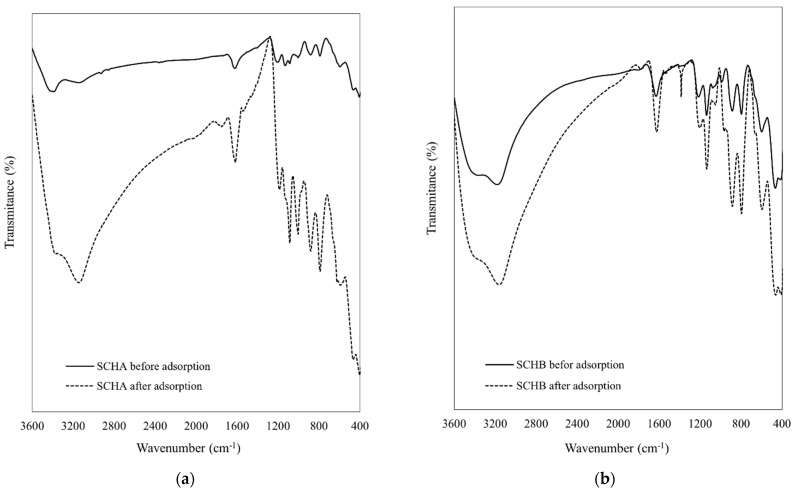
FTIR spectra of synthetic schwertmannite by (**a**) Fe^3+^ hydrolysis (SCH_A_) before and after Cr(VI) adsorption; (**b**) Fe^2+^ oxidation (SCH_B_) before and after Cr(VI) adsorption.

The FTIR spectra of the prepared schwertmannite adsorbents before and after Cr(VI) adsorption were measured and are shown in Figure 13. Analysis of FTIR spectra allows identification of the probable mechanism of Cr(VI) adsorption. Both samples (SCH_A_ and SCH_B_) before adsorption of Cr(VI) had absorption bands in the range 3200–3400 cm^−1^ and 1630 cm^−1^ corresponding to −OH and H_2_O vibrations, respectively. Most of the bands allowing the identification of schwertmannite on the FTIR spectrum are located in the range 1200–600 cm^−^^1^. In this region, the dominant absorption bands are the −SO_4_^2^^−^ stretching vibrations at 980, 1020, 1120, and 1200 cm^−^^1^ and the bending vibration at 600 cm^−^^1^, which are specific sulphate absorption bands for schwertmannite [22]. The −OH absorption bands at 790 and 880 cm^−^^1^ are present in SCH_A_ and SCH_B_, which may indicate low goethite content in both SCH_A_ and SCH_B_. Moreover, a wide band at the wavenumber of about 485 cm^−^^1^ indicates the presence of FeO bonds in the studied adsorbents.

In the FTIR spectra of SCH_A_ and SCH_B_ adsorbents after Cr(VI) adsorption, an increase in the intensity of the peaks at 3300 cm^−^^1^, 1630 cm^−^^1^, and in the range of 800–1200 cm^−^^1^ can be observed. This change may indicate the involvement of −SO_4_^2−^ groups in Cr(VI) adsorption. These results are in line with other studies, which showed that many groups, especially sulphate groups, were responsible for Cr(VI) removal. The appearance of bands between 750 and 950 cm^−^^1^ indicates Cr(VI) ions in the studied samples. In addition, other authors also note that bands from CrO_4_^2−^ appear in this range at 761, 777, 781, 828, 833, 882, 901, and 933 cm^−^^1^ [42,77,78]. In this range, the changes that occurred after adsorption of Cr(VI) onto schwertmannite are most evident. In addition, a new band at the wavenumber of 1380 cm^−^^1^ can be distinguished in the FTIR spectrum. The appearance of a new peak may suggest that a change in the structure of schwertmannite occurred during Cr(VI) adsorption. The vibrations at the wavenumber of 1380 cm^−^^1^ may originate from chromium compounds, e.g., chromium(VI) oxide or chromium(III) sulphate hydrate (Spectral Database for Organic Compounds, SDBS), but Tinti and co-workers indicate that a band approximately 1380 cm^−^^1^ would be due to coordinatively bound water [79].

SEM images of schwertmannite after Cr(VI) adsorption were also taken to determine the effect of adsorption on the structure of the adsorbents used. No significant changes in the morphology of schwertmannite after Cr(VI) adsorption were observed, and therefore these SEM micrographs are not included in this paper. In addition, Regenspurg did not observe any apparent effect on the structure and arrangement of the crystals in the samples containing chromates [42].

**Figure 14:**
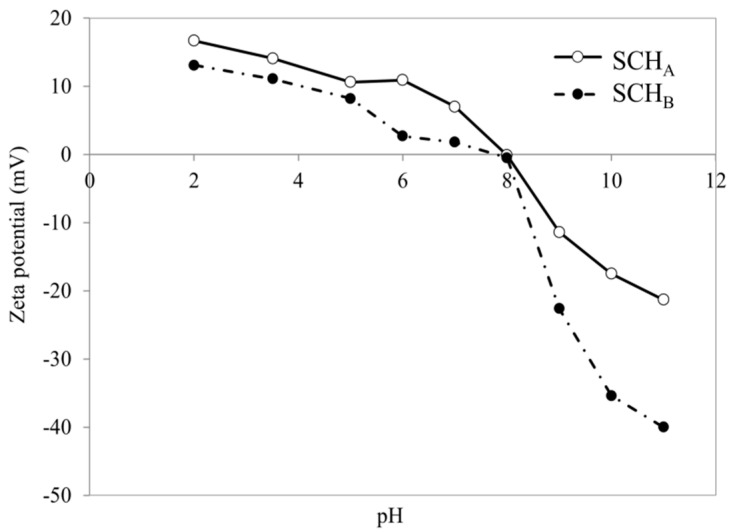
Zeta potential of synthetic schwertmannite adsorbents after Cr(VI) adsorption.

The electrokinetic potential and pH_iep_ of schwertmannite after Cr(VI) adsorption were also determined. The results are shown in Figure 14. After Cr(VI) adsorption, the pH_iep_ of both adsorbents increased and is around 8.0. This confirms the results of Regenspurg that adsorption of Cr(VI) on schwertmannite results in the replacement of sulphate groups with Cr(VI) ions and thus an increase in pH_iep_ compared to the pH_iep_ of schwertmannite before adsorption [42].

After analysing the obtained experimental results (FTIR spectra before and after adsorption, SEM images and electrokinetic potential) and the literature data, it can be concluded that Cr(VI) adsorption on schwertmannite can be controlled in two stages. Khamphila and co-workers found that the adsorption of Cr(VI) on easily accessible active sites during the fast initial stage is likely to be ion exchange with SO_4_^2^^–^ groups. In contrast, the second stage involves the slow diffusion of Cr(VI) from the surface sites into the interior of the adsorbent and the formation of complexes [25]. According to Zhang and co-workers, two types of surface complexes can form on the schwertmannite surface: monodentate (≡FeOCrO_3_^−^) and bidentate (≡Fe_2_O_2_CrO_2_) [52].

## 3. Materials and Methods

### 3.1. Preparation of Schwertmannite Sorbents

The synthetic schwertmannite was obtained by two methods. The first synthesis method (Fe^3+^ hydrolysis) involved the hydrolytic transformation of iron chloride in the presence of large amounts of sulphate ions, according to the method published by Bigham and later by Schwertmann and Cornell [80,81]. For this purpose, 10.8 g FeCl_3_ × 6H_2_O and 3 g Na_2_SO_4_ were added to 2 dm^3^ of deionised water heated to 60 °C in a water bath. The precipitated brown-red suspension was kept at this temperature for 12 min, and it was stirred from time to time. The mixture was then cooled to room temperature and decanted to obtain a concentrated sludge. The sludge was then dialysed for about 30 days. Dialysis was completed when the conductivity of dialysate reached 2–3 μS·cm^−^^1^. The suspension prepared in this way was stored in a glass vessel. The product thus obtained was labelled as synthetic schwertmannite A (SCH_A_).

The second type of schwertmannite was obtained by forced oxidation of iron(II) sulphate, according to the method (Fe^2+^ oxidation) published by Regenspurg and Peiffer [22,52]. In this method, 18.3 g FeSO_4_ × 7H_2_O was dissolved in 1 dm^3^ of deionised water and then 5 cm^3^ 30% H_2_O_2_ was added. The precipitated brown-red suspension was stirred for about 24 h, centrifuged, and then dialysed for about 30 days. Dialysis was completed when the conductivity of dialysate reached 2–3 µS·cm^−^^1^. The suspension prepared in this way was stored in a glass vessel. The obtained material was labelled as synthetic schwertmannite B (SCH_B_).

### 3.2. Characterisation of Schwertmannite

The images were taken using an Axio Imager.M1m optical microscope (Zeiss, Jena, Germany) and a JSM-6610LV scanning electron microscope (JEOL Ltd., Akishima, Japan) after sputtering the samples with carbon using an automatic coating machine JEC-530 (JEOL Ltd., Akishima, Japan). In addition, the dry matter content was measured in both suspensions. Particle size analysis by laser diffraction was carried out using a particle size analyser Mastersizer 2000 (Malvern Instruments Ltd., Malvern, UK). The specific surface areas of schwertmannite A (SCH_A_) and schwertmannite B (SCH_B_) were determined using FlowSorb 2300 apparatus (Micromeritics Instruments Corp, Norcross, GA, USA). The density of schwertmannite sorbents was performed using a pycnometer. The isoelectric point (pH_iep_) was determined by electrophoretic zeta potential measurements using a Zetasizer 2000 (Malvern Instruments Ltd., Malvern, UK). FT-IR measurements of the obtained schwertmannite sorbents were performed with a Vertex 70/70V spectrometer (Brucker, Billerica, MA, USA).

### 3.3. Adsorption Experiments

Potassium dichromate (K_2_Cr_2_O_7_) was used as the source of Cr(VI). A stock solution was prepared in deionised water by dissolving 2.8300 g of potassium dichromate in 1000 cm^3^. The solution had a concentration of 1000 mg·dm^−^^3^ and a pH of about 4.5. The solutions used in the experiments were prepared from the stock solution by diluting it with deionised water. Most of the adsorption studies were performed using a Cr(VI) solution with a concentration of 100 mg·dm^−^^3^. This concentration can be found in waters located in the vicinity of electroplating plants.

The adsorption studies were carried out by a batch method at room temperature. Concentrations of Cr(VI) were determined by measuring the absorbance at the characteristic wavelength (545 nm) using a UV-visible spectrophotometer Evolution 201 (Thermo Fisher Scientific, Madison, WI, USA), accordingly to the diphenylcarbazide method.

In all adsorption studies, schwertmannite suspensions were used directly after synthesis rather than dried material. The volume of the suspension samples was recalculated to dry schwertmannite content. Each experiment was repeated three times under the same conditions. The dry matter content in 1 cm^3^ of the suspension was 8.00 mg and 31.0 mg for SCH_A_ and SCH_B_, respectively. It can be observed that the dry matter content for SCH_B_ is approximately four times higher than for SCH_A_, which significantly affects the adsorption results.

Studies of Cr(VI) adsorption were carried out for both schwertmannite A (SCH_A_) and schwertmannite B (SCH_B_). Removal of Cr(VI) was tested in batch studies as a function of adsorbent dosage, pH, contact time, and initial Cr(VI) concentration. All adsorption studies were carried out using the batch method at room temperature for 24 h. The effect of pH was investigated by adjusting the pH of Cr(VI) solutions using 0.1 M HCl and 0.1 M NaOH. In this set of experiments, 1 cm^3^ of adsorbent (SCH_A_ or SCH_B_) per 10 cm^3^ of 100 mg·dm^−^^3^ Cr(VI) standard solution were taken. The effect of the adsorbent dose was studied by adding the varying volume of adsorbent (SCH_A_ or SCH_B_) from 0.05 to 1 cm^3^ to Cr(VI) solution at an initial concentration of 100 mg·dm^−^^3^ The kinetic experiments were conducted in batch mode by shaking 1 cm^3^ of adsorbent (SCH_A_ or SCH_B_) with Cr(VI) solution at a constant pH (~4.5). Cr(VI) concentration was monitored for 24 h. The adsorption isotherms were performed in two measurement series—in the range of low initial Cr(VI) concentration (1–100 mg·dm^−^^3^) and in the range of high initial Cr(VI) concentration (10–1000 mg·dm^−^^3^). The adsorbent SCH_A_ or SCH_B_ (1 cm^3^) was added to 10 cm^3^ Cr(VI) solution (1–1000 mg·dm^−^^3^). The pH of the solutions was 4.5 and the equilibrium time for this experiment was 24 h.

The amount of Cr(VI) adsorbed (removal in percentages) is calculated as follows (Equation (10)) [28]:

R(%) = ((c_0_ − c)/c_0_)·100 (10)

where c_0_ and c are the initial and the final concentrations of Cr(VI) in the aqueous solution (mg·dm^−3^).

The equilibrium adsorption capacity was calculated from the standard equation (Equation (11)) [28]:

q_e_ = (c_0_ − c)·V/m (11)

where c_0_ and c (mg·dm^−^^3^) are the initial and equilibrium Cr(VI) concentrations, respectively; V (cm^3^) is the volume of the solution and m (g) is the amount of adsorbent.

## 4. Conclusions

The sorption of Cr(VI) from aqueous solutions has been examined on two types of schwertmannite sorbents (SCH_A_ and SCH_B_). SCH_A_ was formed by Fe^3+^ hydrolysis and SCH_B_ by Fe^2+^ oxidation. Based on the experimental results and theoretical predictions, the following conclusions can be drawn:

- Compared with literature data, both SCH_A_ and SCH_B_ are efficient adsorbents for Cr(VI) removal.
- SCH_A_ has a higher adsorption capacity than SCH_B_, despite its smaller specific surface area.
- The well-developed specific surface area suggests that schwertmannite (SCH_A_ and SCH_B_) can be a good Cr(VI) sorbents, especially in acidic conditions.
- Adsorption of Cr(VI) on schwertmannite sorbents should be carried out at pH of around 4 as the removal of Cr(VI) is the most efficient under those conditions.
- The IPD model suggested that intraparticle diffusion is not the only rate-limiting step in Cr(VI) adsorption on schwertmannite.
- The PSO model describes the adsorption kinetics of Cr(VI) on SCH_A_ and SCH_B_ better than the PFO model.
- The sorption isotherm was well described by Freundlich and Langmuir models.
- According to the Langmuir model, the maximum adsorption capacity of Cr(VI) at low initial concentration is 42.97 and 17.54 mg·g^−^^1^ for SCH_A_ and SCH_B_, respectively, and at high initial concentrations is 201.8 and 131.8 mg·g^−^^1^ for SCH_A_ and SCH_B_, respectively.

Although schwertmannite sorbents have been thoroughly investigated for the adsorption of arsenic oxyanions, Cr(VI) removal on schwertmannite is still a poorly explored area. Nevertheless, the results of the present work and those of previously published studies indicate that schwertmannite has promising properties as a Cr(VI) removal sorbent and is worthy of thorough investigation.

## Figures and Tables

**Table 1:** List of adsorption kinetics models used in this work.

Model	Equation	Parameters	Relationship	Reference
Pseudo-first-order (PFO)	log(q_e_ − q_t_) = log(q_1_) − (k_1_/2.303)·t (2)	q_1_ (mg·g^−1^) k_1_ (min^−1^)	log(q_e_ − q_t_) vs. t	[53,54]
Pseudo-second-order (PSO)	(t/q_t_) = (1/(k_2_·q_2_^2^)) + (1/q_2_)·t (3)	q_2_ (mg·g^−1^) k_2_ (g·mg^−1^·min^−1^)	t/q_t_ vs. t	[55,56]
Intraparticle diffusion (IPD)	q_t_ = k_IPD_·t^1/2^ + B (4)	k_IPD_ (mg·g^−1^·min^−1/2^) B (mg·g^−1^)	q_t_ vs. t^1/2^	[57,58]
Liquid film diffusion (LFD)	ln(1 − q_t_/q_e_) = − k_LFD_·t (5)	k_LFD_ (min^−1^)	ln(1 − q_t_/q_e_) vs. t	[58,59]

**Table 2:** Kinetic parameters of pseudo-first-order (PFO) and pseudo-second-order (PSO) models.

Adsorbent		PFO				PSO			
	q_exp_ (mg·g^−1^)	k_1_ (min^−1^)	q_1_ (mg·g^−1^)	R^2^	SD	k_2_ (g·mg^−1^·min^−1^)	q_2_ (mg·g^−1^)	R^2^	SD
SCH_A_	39.98	0.0043	11.49	0.949	20.1	0.0026	39.97	0.999	0.007
SCH_B_	17.69	0.0046	7.49	0.964	7.21	0.0007	17.72	0.999	0.022

**Table 3:** Parameters of the intraparticle diffusion (IDP) and liquid film diffusion (LFD) models.

Adsorbent	IPD						LFD	
	k_IPD1_ (mg·g^−1^·min^−0.5^)	B_1_ (mg·g^−1^)	R^2^	k_IPD2_ (mg·g^−1^·min^−0.5^)	B_2_ (mg·g^−1^)	R^2^	k_LFD_ (min^−1^)	R^2^
SCH_A_	0.1458	30.04	0.957	0.0084	34.88	0.798	0.0041	0.991
SCH_B_	0.0587	12.14	0.907	0.0057	14.30	0.776	0.0044	0.981

**Table 4:** List of adsorption isotherm models used in this work.

Isotherm Model	Equation	Parameters	Reference
Freundlich	q_e_ = k_F_·c_e_^1/n^ (6)	k_F_ ((dm^3^)^1/n^·mg^(1−1/n)^·g^−1^) n (−)	[63,64]
Langmuir	q_e_ = (q_L_·k_L_·c_e_)/(1 + k_L_·c_e_) (7)	q_L_ (mg·g^−1^) k_L_ (dm^3^·mg^−1^)	[64,65]
Temkin	q_e_ = (R·T/B_T_)·ln(k_T_·c_e_) (8)	B_T_ (kJ·mol^−1^) k_T_ (dm^3^·g^−1^)	[64,66]

**Table 5:** Parameters of isotherm adsorption models.

	Low Concentration of Cr(VI) (1–100 mg·dm^−3^)		High Concentration of Cr(VI) (10–1000 mg·dm^−3^)	
	SCH_A_	SCH_B_	SCH_A_	SCH_B_
Freundlich				
n	1.68	1.77	1.03	1.29
1/n	0.595	0.565	0.971	0.775
k_F_ ((dm^3^)^1/n^ mg^(1^^–^^1/n)^·g^−1^)	3.49	2.24	1.91	1.17
R^2^	0.998	0.996	0.985	0.982
Langmuir				
q_L_ (mg·g^−1^)	42.97	17.54	201.8	131.8
k_L_ (dm^3^·mg^−1^)	0.054	0.116	0.0127	0.0055
R^2^	0.984	0.976	0.966	0.961
Temkin				
b_T_ (J·mol^−1^)	244.6	823.7	21.8	56.9
k_T_ (dm^3^·g^−1^)	0.584	3.50	0.0733	0.0395
R^2^	0.887	0.870	0.621	0.788

**Table 6:** Comparison of adsorption capacity of schwertmannite synthesised by different methods for Cr(VI).

Method of Synthesis	q_max_ (mg·g^−1^)	Reference
Fe^2+^ oxidation	17.5 *	This paper
	131.8 **	
	219	[52]
Fe^3+^ hydrolysis	43.0 *	This paper
	201.8 **	
	83.5	[25]
	105	[71]
	178.6	[46]
Biosynthesis	38.8	[26]
	35.3	[72]
	19.0	[73]
	58.2	[74]

* The maximum adsorption capacity according to the Langmuir model calculated for the experiment performed in low concentrations of Cr(VI). ** The maximum adsorption capacity according to the Langmuir model calculated for the experiment performed in high concentrations of Cr(VI).

## Nomenclature

t	time (min)
q_e_	the amount of Cr(VI) adsorbed at equilibrium (mg·g^−1^)
q_t_	the amount of Cr(VI) adsorbed at time t (mg·g^−1^)
c_e_	the equilibrium concentration of Cr(VI) (mg·dm^−3^)
c_0_	the initial concentration of Cr(VI) (mg·dm^−3^)
m	the adsorbent mass (g)
V	the solution volume (dm^3^)
q_1_	the adsorption capacity of Cr(VI) at equilibrium for pseudo-first-order model (mg·g^−1^)
k_1_	the rate constant of pseudo-first-order model (min^−1^)
q_2_	the adsorption capacity of Cr(VI) at equilibrium for pseudo-second-order model (mg·g^−1^)
k_2_	the rate constant of pseudo-second-order model (g·mg^−1^·min^−1^)
k_IPD_	the intraparticle diffusion rate constant (mg·g^−1^·min^−1/2^)
B	the parameter related to the thickness of the boundary layer (mg·g^−1^)
k_LFD_	the external mass transfer coefficient (min^−1^)
k_F_	the Freundlich constant indicative of the relative adsorption capacity of the adsorbent ((dm^3^)^1/n^·mg^(1−1/n)^·g^−1^)
n	the Freundlich equation exponent (-)
q_L_	the maximum adsorption capacity in Langmuir model (mg·g^−1^)
k_L_	the Langmuir constant related to the energy of adsorption (dm^3^·mg^−1^)
R	the universal gas constant (kJ·mol^−1^·K^−1^)
T	temperature (K)
B_T_	the Temkin constant related to heat of sorption (kJ·mol^−1^)
k_T_	the Temkin equilibrium isotherm constant (dm^3^·g^−1^)
R_L_	the separation parameter (-)
q	the amount of Cr(VI) adsorbed (mg·g^−1^)
c	the concentration of Cr(VI) (mg·dm^−3^)
